# Modifying effect of gender on the prognostic value of clinicopathological factors and Ki67 expression in melanoma: a population-based cohort study

**DOI:** 10.1186/2042-6410-3-16

**Published:** 2012-07-02

**Authors:** Marie Fridberg, Liv Jonsson, Julia Bergman, Björn Nodin, Karin Jirström

**Affiliations:** 1 Department of Clinical Sciences, Division of Pathology, Lund University, Skåne University Hospital, 221 85 Lund, Sweden; 2 Department of Immunology, Genetics and Pathology, Uppsala University, 751 85 Uppsala, Sweden

**Keywords:** Sex, Malignant melanoma, Clinicopathological characteristics, Prognosis

## Abstract

**Background:**

Malignant melanoma is the most deadly form of skin cancer. Female sex is known to have a protective effect on incidence, tumour characteristics, and mortality from melanoma. However, the potentially modifying effect of sex on the prognostic significance of clinicopathological and investigative factors is generally not taken into consideration in biomarker studies. In this study, we compared the sex-specific distribution and prognostic value of established tumour characteristics and Ki67 expression in 255 cases of incident primary melanoma in a prospective, population-based cohort study.

**Methods:**

The study included 255 incident cases of melanoma, 132 females and 123 males, in the Malmö Diet and Cancer Study. Tumours from 226 (88.6%) cases had been assembled in tissue microarrays. Clinicopathological factors and immunohistochemical Ki67 expression were assessed and correlated with disease-free survival (DFS) and overall survival (OS) using Kaplan-Meier analysis, log rank test and univariable and multivariable Cox regression analyses, stratified for gender. Effect of gender on melanoma-specific survival (MSS) after first recurrence was also analysed.

**Results:**

Women were significantly younger at diagnosis than men (p = 0.012). The most common tumour sites were the legs in women (37.5%) and the dorsal trunk in men (37.8%). Kaplan-Meier analysis revealed that tumour location had no prognostic impact in women, but in men, location to the frontal trunk was significantly associated with a reduced DFS compared with all other locations combined and location to the dorsal trunk was significantly associated with a prolonged OS. High Ki67 expression was significantly associated with a reduced DFS and OS in men but not in women, also when adjusted for other factors. In men, but not in women, ulceration was an independent prognostic factor for both DFS and OS. MSS after first local, regional or distant recurrence was significantly shorter for men than for women.

**Conclusions:**

The results from this study demonstrate that the prognostic value of tumour location, Ki67 expression and ulceration in melanoma differs according to gender. These findings need to be validated in future studies, as they may help improve prognostication in patients with melanoma. Moreover, our findings demonstrate that sex-stratified analyses add valuable information to biomarker studies.

## Background

Malignant melanoma is an aggressive form of cancer with an increasing incidence and mortality worldwide [1]. Once a patient has moved into the stage of generalized disease, survival is very poor [2,3], but the clinical course of melanoma is highly variable even in patients with thin melanomas and localized disease [4-6]. Despite increasing insights into melanoma biology and advances in various “omics” technologies [7-9], no prognostic biomarkers have yet been incorporated into clinical protocols. One reason for the difficulties in bridging this translational gap might be that, while a protective effect of female gender on incidence, tumour characteristics, and mortality from melanoma is evident [10,11], the prognostic value of putative biomarkers is generally not evaluated differently in women and men. The negative association of male gender with survival from melanoma cannot solely be attributed to the larger proportion of tumours with unfavourable clinicopathological characteristics, since gender remained prognostic in several studies, also when adjusted for these factors [2,10,12,13]. Moreover, women have been demonstrated to have a prolonged survival compared to men even after development of distant metastasis [14]. Taken together, it is evident that hormonal and other sex-related factors affect the clinical course of the disease and, therefore, sex-stratified analyses might add important information to studies aiming at identifying novel prognostic and treatment predictive biomarkers in melanoma. Biomarker studies in other cancer forms affecting both sexes, e.g. colorectal cancer, have indeed revealed a modifying effect of sex on the prognostic value of certain markers [15,16].

In the present study, we examined the distribution and prognostic value of clinicopathological characteristics of melanoma, including Ki67 expression as an investigative marker, in men and women, respectively. To this end, we analysed 255 cases of incident malignant melanoma in the prospective, population-based cohort Malmö Diet and Cancer Study [17,18].

## Methods

### Patients

Until end of follow-up 31 December 2008, 264 incident cases of malignant melanoma had been registered in the prospective, population-based cohort study Malmö Diet and Cancer Study (MDCS)[17]. Cases were identified from the Swedish Cancer Registry up until 31 Dec 2007, and from The Southern Swedish Regional Tumour Registry for the period of 1 Jan-31 Dec 2008. Nine (3.4%) cases for whom clinical and pathology records were missing were excluded from the study, leaving 255 cases available for analysis. All tumours with available slides and/or paraffin blocks were histopathologically re-evaluated on haematoxylin and eosin stained slides whereby information on lymphocytic infiltration (none, mild, moderate or brisk), ulceration (absent or present), mitotic count and vascular invasion was obtained. Data on location, Clark level and Breslow depth of invasion was obtained from the clinical- and/or pathology records. Information on recurrence (local, regional or distant) was obtained in 2010 from patient records and pathology reports. Information on vital status and cause of death was obtained from the Swedish Cause of Death Registry up until 31 Dec 2009. Follow-up started at date of diagnosis and ended at death, emigration or 31 Dec 2009, whichever came first. Median follow-up time was 6.84 years (range 0.64-17.05) for the full cohort (n = 255) and 7.29 years (range 1.10-17.05) for patients alive (n = 202). Patient and tumour characteristics of the cohort have been described in detail previously [18]. Ethical permission was obtained from the Ethics Committee at Lund University for the MDCS (Ref. 51/90), and the present study (Ref. 530/2008).

### Tissue microarray construction and immunohistochemistry

Paraffin-embedded tumour specimens were collected from the archives of the pathology departments in the region of Skåne in southern Sweden. Tumours with an insufficient amount of material were excluded whereby 226/255 (88.6%) cases were suitable for TMA construction. Areas representative of cancer were then marked on haematoxylin & eosin stained slides and TMAs constructed as previously described. In brief, three 0,6 mm cores were taken from each tumour and mounted in a new recipient block using semi-automated arraying device (TMArrayer, Pathology Devices, Westminster, MD, USA). The distribution of clinicopathological characteristics was similar in tumours included in the TMA cohort (total n = 226) and tumours not suitable for TMA construction (total n = 29), except for histological subtype, with no tumours being denoted as nodular in the latter category (0/23 vs 53/225, p = 0.005) but an equal distribution of the other subtypes (data not shown).

For immunohistochemical analysis, 4 um TMA-sections were automatically pre-treated using the PT-link system (DAKO, Glostrup, Denmark) and then stained in an Autostainer Plus (DAKO) using a monoclonal anti- Ki67 antibody (MIB-1, DAKO, diluted 1:200). Ki67 expression was annotated as the fraction of positive staining cells and denoted as 0 (0-1%), 1(2-25%) and 2(>25%). To check for heterogeneity, Ki67 expression was also analysed on full-face sections from 26 cases representing tumours with varying thickness.

### Statistical analysis

Chi-square, Spearman´s Rho and Mann Whitney U tests were used for comparison of the distribution of clinicopathological characteristics in the full cohort and according to gender. Disease-free survival (DFS) time was determined from the date of diagnosis of the primary melanoma to the date of diagnosis of the first local, regional or distant recurrence or death from malignant melanoma. Overall survival (OS) was assessed by calculating the risk of death from all causes, overall mortality. Follow-up started at date of diagnosis and ended at death, emigration or 31 Dec 2009, whichever came first. Melanoma- specific survival (MSS) time was calculated for patients who developed recurrent disease. Follow-up started at date of diagnosis and ended at recurrent disease, death, loss to follow-up (emigration) or last date of follow-up with regard to recurrent disease. No recurrences were recorded following the last date of follow-up regarding death, i.e. 31 Dec 2009. Follow-up started at date of diagnosis and ended at death, emigration or 31 Dec 2009, whichever came first. Kaplan-Meier analysis and log rank test were used to illustrate differences in DFS, OS and MSS. Cox regression proportional hazards models were used to estimate the impact of the investigated parameters on survival in both uni- and multivariable analysis. Co-variates were entered into the multivariate analysis using backward selection. The prognostic interaction between the clinicopathological factors and gender was explored by a Cox model including the interaction variable. All calculations were performed using IBM SPSS Statistics Version 20 (SPSS Inc, Chicago, IL). All statistical tests were two-sided and a p value < 0.05 was considered statistically significant.

## Results

### Distribution of clinicopathological characteristics according to gender

The distribution of clinicopathological characteristics according to gender are shown in Table 1. Women were significantly younger at diagnosis than men (p = 0.012). There was also a sex-related difference in tumour location, with the legs being the most common location in women (39.5% vs 10.9% in men) and the dorsal trunk being the most common location in men (37.8% vs 15.5% in women). Following antibody optimisation and staining, Ki67 expression could be evaluated in 206/226 (91.1%) cases. There was an excellent concordance between Ki67 expression on full-face sections and corresponding TMA cores. There was no significant difference in Ki67 expression between the sexes. All other established clinicopathological factors were evenly distributed among both sexes (Table 1).

**Table 1:** Distribution of clinicopathological parameters in women and men

**n (% for columns)**	**Female**	**Male**	**p-value**
	**n = 132 (51.8)**	**n = 123 (48.2)**	
**Age**			0.012*
Mean	65	69	
Median	65	70	
Range	46-84	53-80	
**Location**			0.001**
Dorsal trunk	20 (15.5)	45 (37.8)	
Frontal trunk	15 (11.6)	19 (16.0)	
Arms	29 (22.5)	18 (15.1)	
Legs	51 (39.5)	13 (10.9)	
Head and neck	14 (10.9)	24 (20.2)	
Unknown	3	4	
**Histological type**			0.372
SSM	80 (62.5)	80 (66.7)	
NMM	27 (21.1)	26 (21.7)	
LMM	16 (12.5)	13 (10.8)	
Other	5 (3.9)	1 (0.8)	
Unknown	4	3	
**Breslow thickness (mm)**			0.417
Mean	1.44	1.72	
Median	0.68	0.80	
Range	(0.11-16.00)	(0.08-40.00)	
Unknown	4	3	
**Thickness (AJCC)**			0.180
< = 1 mm	86 (67.2)	72 (60.0)	
1.1-2 mm	19 (14.8)	17 (14.2)	
2.1-4 mm	17 (13.3)	23 (19.2)	
>4 mm	6 (4.7)	8 (6.7)	
Unknown	4	3	
**Clark level**			0.999
II	46 (36.2)	47 (39.2)	
III	57 (44.9)	46 (38.3)	
IV	21 (16.5)	23 (19.2)	
V	3 (2.4)	4 (3.3)	
Unknown	5	3	
**Clinical stage**			0.135
1A-B	98 (89.9)	81 (82.7)	
2A-B	9 (8.3)	15 (15.3)	
3-4	2 (1.8)	2 (2.0)	
Unknown	23	25	
**Ulceration**			0.962
No	111(86.0)	103 (85.8)	
Yes	18 (14.09)	17 (14.2)	
Unknown	3	3	
**Vascular invasion**			0.076
No	123 (96.9)	109 (91.6)	
Yes	4 (3.1)	10 (8.4)	
Unknown	5	4	
**Mitotic count**			0.769
0/mm^2^	69 (52.7)	62 (50.8)	
> = 1/mm^2^	62 (47.3)	60 (49.2)	
Unknown	3	1	
**Lymphocytic infiltrate**			0.815
None-mild	38 (29.7)	34 (28.3)	
Moderate-brisk	90 (70.3)	86 (71.7)	
Unknown	4	3	
**Ki 67 expression**			0.133
0-1%	4 (3.9)	11 (10.7)	
2-25%	73 (70.9)	71 (68.9)	
>25%	26 (25.2)	21 (20.4)	
Unknown	29	20	
**Local, regional or distal recurrence**			0.360
No	111 (84.1)	98 (79.7)	
Yes	21 (15.9)	25 (20.3)	
**Vital status**			
Alive	114 (86.4)	88 (71.5)	
Dead	18 (13.6)	35 (28.5)	0.004**
Dead from melanoma	9 (6.8)	19 (15.4)	0.028*

The p-values refer to comparisons of male and female tumours. Mann–Whitney test for comparison of medians and Chi square test for X × 2 tables. The categories marked as not done and unknown were not included in the statistical analysis.

### Prognostic value of clinicopathological characteristics in women and men

**Figure 1:**
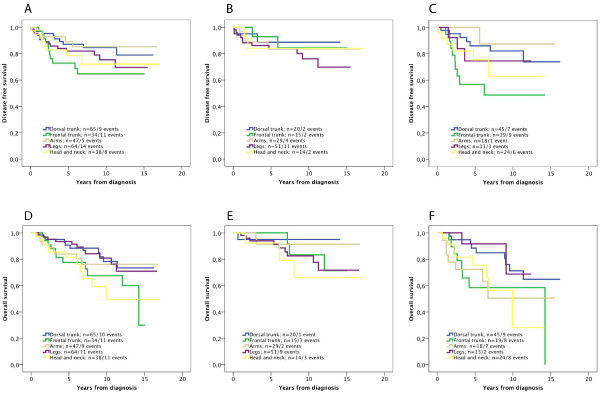
** Kaplan-Meier estimates of the impact of tumour location and disease free and overall survival in the full cohort, women and men.** Kaplan-Meier analysis of disease free survival according to tumour location in (**A**) the full cohort, (**B**) women and (**C**) men (logrank p-values 0.228, 0.789 and 0.031, respectively), and overall survival according to tumour location in (**D**) the full cohort, (**E**) women and (**F**) men (logrank p-values 0.134, 0.556 and 0.075, respectively).

Kaplan-Meier analysis revealed no significant difference in the prognostic value of tumour location in the full cohort or in women (Figure 1A-B), while in men, anatomic location was significantly associated with DFS (Figure 1C). Similar trends were seen for OS, but none reached significance (Figure 1 D-F). Univariable Cox regression analysis confirmed the significant association of location to the frontal trunk with a reduced DFS compared to all other locations (HR = 3.06; 95% CI 1.36-6.89) in men, and location to the dorsal trunk with a significantly prolonged OS (HR = 0.44; 95% CI 0.20-0.96) compared to all other locations in men. These associations did however not remain significant in multivariable analysis, adjusted for the other factors (data not shown). Univariable analyses did not reveal any significant associations with DFS or OS for the other locations (data not shown).

Nearly all established prognostic factors had a significant impact on DFS in univariable analysis (Table 2), except age (all categories), sex, tumour location (full cohort and women), lymphocytic infiltration (women) and Ki67 expression (women). After adjustment by all other variables, two factors retained an independent prognostic value in the full cohort; clinical stage and vascular invasion. In women, only vascular invasion remained an independent predictor of a reduced DFS. In men, three factors retained an independent prognostic value; ulceration, vascular invasion, and Ki 67 expression (Table 2).

**Table 2:** Univariable and multivariable analyses of factors associated with disease free survival in the full cohort, women and men

***Disease free survival***	***Univariable***			***Multivariable***		
	**All HR(95%CI)**	**Women HR(95%CI)**	**Men HR(95%CI)**	**All HR(95%CI)**	**Women HR(95%CI)**	**Men HR(95%CI)**
**Age**						
Continuous	1.01 (0.97-1.05)	1.00 (0.95-1.05)	1.01 (0.95-1.07)	0.98 (0.94-1.02)	1.00 (0.94-1.06)	1.00 (0.92-1.09)
**Sex**						
Women	1.00	-	-	1.00	-	-
Men	1.51 (0.85-2.70)	-	-	1.06 (0.52-2.15)	-	-
**Histological subtype**						
LMM + SSM	1.00	1.00	1.00	1.00	1.00	1.00
NMM	5.63 (3.15-10.08)	6.36 (2.67-15.12)	5.39 (2.41-12.09)	1.21 (0.55-2.63)	1.47 (0.48-4.54)	1.49 (0.46-4.82)
**Breslow thickness**						
<=1 mm	1.00	1.00	1.00	1.00	1.00	1.00
> 1 mm	8.44 (4.27-16.68)	12.17 (4.07-36.34)	6.12 (2.14-14.73	0.61 (0.10-3.70)	0.17 (0.01-2.95)	1.45 (0.15-14.22)
**Clark level**						
II-III	1.00	1.00	1.00	1.00	1.00	1.00
IV-V	3.75 (2.11-6.69)	6.28 (2.65-14.89)	2.37 (1.07-5.22)	0.52 (0.26-1.03)	1.04 (0.32-3.43)	0.36 (0.12-1.07)
**Ulceration**						
No	1.00	1.00	1.00	1.00	1.00	1.00
Yes	5.82 (3.13-10.80)	5.90 (2.41-14.42)	7.21 (2.97-17.48)	1.56 (0.65-3.76)	0.70 (0.23-2.12)	5.46 (1.70-17.53)
**Clinical stage**						
1	1.00	1.00	1.00	1.00	1.00	1.00
2-4	15.02 (16.39-35.30)	25.41 (5.81-111.08)	11.76 (3.86-35.87)	5.56 (3.02-24.28)	6.88 (1.64-28.89)	6.39 (1.56-26.25)
**Vascular invasion**						
No	1.00	1.00	1.00	1.00	1.00	1.00
Yes	9.25 (3.56-17.79)	20.48 (5.47-76.65)	6.87 (2.96-15.97)	3.44 (1.63-7.25)	4.79 (1.21-19.01)	11.15 (3.42-36.38)
**Mitotic count**						
0/mm^2^	1.00	1.00	1.00	1.00	1.00	1.00
> = 1/mm^2^	7.95 (3.56-17.80)	27.24 (3.65-203.10)	4.83 (1.92-12.13)	3.28 (1.11-9.73)	6.87 (0.77-61.05)	2.21 (0.53-9.13)
**Lymphocytic infiltrate**						
None-mild	1.00	1.00	1.00	1.00	1.00	1.00
Moderate-brisk	0.43 (0.24-0.78)	0.45 (1.19-1.08)	0.43 (0.19-0.94)	0.73 (0.37-1.44)	0.87 (0.31-2.43)	0.69 (0.25-1.90)
**Ki67 expression**						
0-25%	1.00	1.00	1.00	1.00	1.00	1.00
>25%	2.46 (1.33-4.56)	1.67 (0.67-4.15)	4.06 (1.74-9.48)	1.74 (0.85-3.54)	0.96 (0.24-3.78)	6.14 (1.76-21.39)

LMM = lentigo maligna melanoma, SSM = superficial spreading melanoma, NMM = nodular malignant melanoma.

The relative risk of death (OS) according to the different factors was also calculated, whereby age and male sex were associated with a significantly poorer survival in univariable but not multivariable analysis (Table 3). For the other factors, the associations with OS were similar to DFS in univariable analysis, with lymphocytic infiltration and Ki67 expression not being prognostic in women. After adjustment by all other variables (Table 3), three factors retained an independent prognostic value in the full cohort; nodular histology, ulceration, and vascular invasion. In females, nodular histology and vascular invasion remained independent predictors of a reduced OS. In males, nodular histology, ulceration, vascular invasion and Ki 67 expression remained independent prognostic factors.

**Table 3:** Univariable and multivariable analyses of factors associated with overall survival in the full cohort, women and men

***Overall***	***Univariable***			***Multivariable***		
	**All HR(95%CI)**	**Women HR(95%CI)**	**Men HR(95%CI)**	**All HR(95%CI)**	**Women HR(95%CI)**	**Men HR(95%CI)**
**Age**						
Continuous	1.09 (1.04-1.13)	1.08 (1.02-1.15)	1.08 (1.02--1.14)	1.07 (1.02-1.13)	1.09 (1.02-1.16)	1.10 (1.02-1.18)
**Sex**						
Women	1.00	-	-	1.00	-	-
Men	2.60 (1.47-4.61)	-	-	1.89 (0.95-3.79)	-	-
**Histological subtype**						
LMM + SSM	1.00	1.00	1.00	1.00	1.00	1.00
NMM	3.86 (2.21-6.74)	3.93 (1.54-10.03)	4.17 (2.06-8.44)	2.73 (1.38-5.40)	3.35 (1.25-8.95)	2.88 (1.21-6.84)
**Breslow thickness**						
<=1 mm	1.00	1.00	1.00	1.00	1.00	1.00
> 1 mm	4.17 (2.38-7.32)	4.53 (1.74-11.79)	3.66 (1.83-7.34)	0.73 (0.16-3.42)	0.56 (0.04-7.58)	0.58 (0.08-4.28)
**Clark level**						
II-III	1.00	1.00	1.00	1.00	1.00	1.00
IV-V	2.27 (1.28-4.05)	1.87 (0.66-5.25)	2.22 (1.11-4.44)	0.84 (0.36-1.92)	0.53 (0.15-1.88)	1.10 (0.35-3.41)
**Ulceration**						
No	1.00	1.00	1.00	1.00	1.00	1.00
Yes	6.29 (4.46-11.45)	5.83 (2.14-15.85)	7.33 (3.44-15.62)	2.63 (1.19-5.83)	1.01 (0.18-5.76)	3.34 (1.30-8.60)
**Clinical stage**						
1	1.00	1.00	1.00	1.00	1.00	1.00
2-4	6.48 (3.05-13.73)	5.48 (1.43-21.04)	6.34 (2.49-16.14)	1.71 (0.57-5.08)	2.54 (0.59-10.89)	0.59 (0.11-3.29)
**Vascular invasion**						
No	1.00	1.00	1.00	1.00	1.00	1.00
Yes	4.89 (2.38-4.08)	13.43 (2.84-63.50)	2.82 (1.23-6.50)	4.54 (1.90-10.84)	19.95 (3.63-109.75)	4.89 (1.66-14.41)
**Mitotic count**						
0/mm^2^	1.00	1.00	1.00	1.00	1.00	1.00
> = 1/mm^2^	2.96 (1.66-5.28)	3.54 (1.26-9.96)	2.86 (1.41-5.82)	1.49 (0.65-3.40)	1.63 (0.34-7.81)	1.34 (0.44-4.07)
**Lymphocytic infiltrate**						
None-mild	1.00	1.00	1.00	1.00	1.00	1.00
Moderate-brisk	0.46 (0.26-0.79)	0.53 (0.17-1.09)	0.45 (0.22-0.90)	0.66 (0.35-1.25)	1.03 (0.30-3.49)	0.60 (0.27-1.35)
**Ki67 expression**						
0-25%	1.00	1.00	1.00	1.00	1.00	1.00
>25%	1.97 (1.06-3.64)	1.79 (0.66-4.87)	2.38 (1.09-5.22)	1.55 (0.73-3.30)	1.46 (0.34-6.14)	2.65 (1.08-6.52)

LMM = lentigo maligna melanoma, SSM = superficial spreading melanoma, NMM = nodular malignant melanoma.

We also explored a possible interaction term between sex and each prognostic factor for DFS and OS, but found no significant effect.

The prognostic value of the RNA-binding motif 3 (RBM3) protein, previously demonstrated to be an independent marker of good prognosis in this cohort [18] did not differ according to gender (data not shown).

### Impact of gender on survival after first recurrence

**Figure 2:**
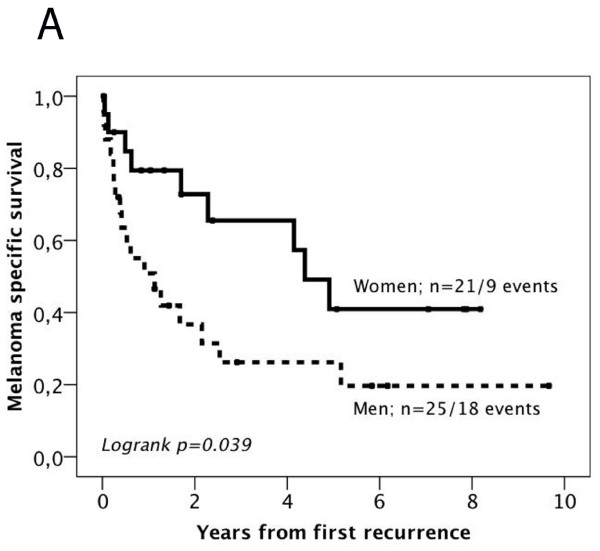
** Kaplan-Meier estimates of the impact of sex on melanoma specific and overall survival after first recurrence.** Kaplan-Meier analysis of (**A**) melanoma-specific and (**B**) 2-year overall survival according to gender after first local, regional or distant recurrence.

In light of previous findings of a more favourable outcome in women after development of distant metastasis [14], we also examined the impact of gender on MSS and 2-year OS after first local, regional or distant recurrence (n = 46). Kaplan Meier analysis revealed a significantly higher mortality from melanoma in men compared to women (p = 0.039) (Figure 2A) as well as a significantly higher mortality after 2 years (p = 0.038) (Figure 2B). The significant association of male gender and shorter survival from melanoma was also confirmed in univariable Cox regression analysis (HR = 2.29; 95% CI = 1.02-5.13, p = 0.044 for MSS and HR = 2.64; 95% CI = 1.02-6.82, p = 0.046 for 2-year OS). For total OS, a similar, however non-significant, trend, was seen (data not shown).

### Associations of Ki67 expression with other clinicopathological factors in the full cohort and according to gender

In light of the differential prognostic impact of Ki67 expression in females and males, we examined whether the association of Ki67 with other tumour and patient characteristics might differ between the sexes. As demonstrated in Table 4, the association of Ki67 and most other factors were similar in both sexes, with the exception of Clark level and ulceration, for which no associations with Ki67 were seen in men, in contrast to a highly significant association in women.

**Table 4:** Associations of Ki67 expression with clinicopathological factors in the full cohort, women and men

***Ki67 expression***	***All***			***Women***			***Men***		
	***Low***	***High***	***P***	***Low***	***High***	***P***	***Low***	***High***	***P***
n(%)	159 (77.2)	47 (22.8)		77 (58.3)	26 (19.7)		82 (79.6)	21 (20.4)	
**Age**									
Continuous	68 (46–81)	70 (47–83)	0.233	66 (46–81)	69 (47–83)	0.366	70 (53–80)	72 (55–80)	0.317
**Location**									
Dorsal trunk	43 (28.1)	10 (21.3)	0.120	13 (17.3)	3 (11.5)	0.135	30 (38.5)	7 (33.3)	0.520
Frontal trunk	19 (12.4)	4 (8.5)		7 (9.3)	1 (3.8)		12 (15.4)	3 (14.3)	
Arms	27 (17.6)	9 (19.1)		17 (22.7)	6 (23.1)		10 (12.8)	3 (14.3)	
Legs	44 (28.8)	27.7)		33 (44.0)	11 (42.3)		1 (14.1)	2 (9.5)	
Head and neck	20 (13.1)	11 (23.4)		5 (6.7)	5 (19.2)		15 (19.2)	6 (28.6)	
*Unknown*	6	0		2	0		4	0	
**Histological subtype**									
SSM, LMM, Other	130 (82.3)	24 (51.1)	<0.001	63 (81.8)	14 (53.8)	0.004	67 (82.7)	10 (47.6)	0.001
NMM	28 (17.7)	23 (48.9)		14 (18.2)	12 (46.2)		14 (17.3)	11 (52.4)	
*Unknown*	1	0		0	0				
**Breslow thickness**									
<=1 mm	107 (68.2)	15 (31.9)	<0.001	54 (71.1)	9 (34.6)	0.001	53 (65.4)	6 (28.6)	0.002
>1 mm	50 (31.8)	32 (68.1)		22 (28.9)	17 (65.4)		28 (34.6)	15 (71.4)	
*Unknown*	*2*	*0*		*0*	*0*		*1*	*0*	
**Clark level**									
II-III	128 (81.5)	28 (60.9)	0.004	64 (84.2)	14 (56.0)	0.004	64 (79.0)	14 (66.7)	0.237
IV-V	29 (18.5)	18 (39.1)		12 (15.8)	11 (44.0)		17 (21.0)	7 (33.3)	
*Unknown*	2	1		2	1		1	0	
**Ulceration**									
No	142 (89.9)	31 (66.0)	<0.001	70 (90.9)	15 (57.7)	<0.001	72 (88.9)	16 (76.2)	0.134
Yes	16 (10.1)	16 (34.0)		7 (9.1)	11 (42.3)		9 (11.1)	5 (23.8)	
*Unknown*	1	0		0	0		1	0	
**Clinical stage**									
1	121 (76.1)	20 (42.6)	<0.001	60 (88.2)	13 (81.2)	0.459	61 (91.0)	7 (43.8)	<0.001
2-4	14 (8.8)	12 (25.5)		8 (11.8)	3 (18.8)		6 (9.0)	9 (56.2)	
*Unknown*	24	15		9	10		15	5	
**Vascular invasion**									
No	146 (91.8)	46 (97.9)	0.149	73 (94.8)	26 (100.0)	0.238	73 (89.0)	20 (95.2)	0.393
Yes	13 (8.2)	1 (2.1)		4 (5.2)	0		9 (11.0)	1 (4.8)	
*Unknown*	0	0		0	0		0	0	
**Mitotic count**									
0/mm^2^	88 (55.3)	13 (27.7)	0.001	42 (54.5)	7 (26.9)	0.015	46 (56.1)	6 (28.6)	0.025
> = 1/mm^2^	71 (44.7)	34 (72.3)		35 (45.5)	19 (73.1)		36 (43.9)	15 (71.4)	
*Unknown*	0	0		0	0		0	0	
**Lymphocytic infiltrate**									
None-mild	42 (26.4)	14 (29.8)	0.667	20 (26.0)	08 (30.8)	0.636	22 (27.2)	6 (28.6)	0.898
Moderate-brisk	116 (73.0)	33 (70.2)		57 (74.0)	18 (69.2)		59 (72.8)	15 (71.4)	
*Unknown*	1	0		0	0		1	0	

P-values refer to Mann Whitney *U* test for comparison of means and Chi square test for X × 2 tables. The categories marked as unknown were not included in the analyses.

## Discussion

In this study, we have demonstrated several sex-related differences in the distribution and prognostic value of patient and tumour characteristics in malignant melanoma. The significant difference in tumour location between the sexes, with a predilection for tumours located to the extremities in women and the trunk in men, is in line with the expected [11,13,14]. Interestingly, though, tumour location was not prognostic in women, in contrast to men, where melanomas located to the frontal trunk had a significantly reduced DFS. Several studies have reported a worse prognosis for melanomas located to the trunk [19-22], and Balzi et al found a significantly improved prognostic advantage for females over males with lesions located on the trunk [14], which is in line with our findings. It is however noteworthy that, in our study, where location to the dorsal and frontal trunk were denoted as separate categories, DFS was significantly poorer for men having melanomas located to the frontal trunk compared to the dorsal trunk. A similar trend was seen in the full cohort, but not in women. One explanation for trunk melanomas having a worse prognosis might be that they often drain to multiple lymph node basins [23], but the reason for the here observed discrepant outcome between melanomas located to the frontal and dorsal trunk in males, evident for both DFS and OS, remains unclear and should be considered in future studies related to the prognostic significance of tumour site.

The MDCS is a population-based cohort study, and, therefore, a potential selection bias compared to the general population must be considered [24]. Since participants were > = 45 years at baseline, mean age at diagnosis was higher than in the average population. Hence, in light of the fact that high age is often associated with more advanced melanoma [10], the comparatively low proportion of cases with advanced melanoma is somewhat unexpected, and might indicate an increased awareness and tendency to seek medical attention earlier among study participants. In our study, women were significantly younger at diagnosis than men. Although the life-time risk of melanoma is higher in males than in females, a reversed tendency is seen in adolescents and young adults, where the rate of increase of the incidence of melanoma is higher in females than in males [25,26]. This might be explained by the fact that intentional tanning, not least the use of tanning beds, is more frequent in younger women [27-29]. Unfortunately, information on tanning habits is not available for participants in the MDCS.

The observation that Ki67 expression was a significant predictor of an adverse clinical outcome in men but not in women, also when adjusted for other factors, is intriguing. Notably, gender had no modifying effect on the prognostic value of mitotic index, another tumour characteristic reflecting tumour proliferation, and Breslow thickness had a similar impact on survival in both sexes. A prognostic role for Ki67 expression is supported by several studies [30-33], and, moreover, the rate of proliferation has been demonstrated to decrease when melanoma cells enter the dermis, corresponding to the transition from *in situ* to invasive radial growth, and to increase again with the onset of the vertical growth phase [33]. Information on growth phase was not available for the patients in our study, and Ki67 expression was assessed according to the estimated proportion of all melanoma cells, without further fine-tuning according to lesional compartment, since this would have required analysis of full-face sections. Sex-related differences have been demonstrated in the time course and pattern of melanoma metastasis [14], and our findings also indicate a potential influence of sex hormones on the balance between invasion and proliferation in the earlier phases of melanoma progression. Notably, there was no significant difference between sexes in the distribution or associations of mitotic index, tumour thickness and Ki67 expression, that might explain the differential prognostic impact of the latter in women and men. There were however sex-related differences in the associations of Ki67 expression with ulceration and Clark level, both being strongly associated with Ki67 expression in women but not in men. Moreover, in men, but not in women, ulceration was an independent prognostic factor for both DFS and OS. Of note, ulcerated melanoma has been proposed to constitute a biologically distinct subtype of melanoma [34], and, hence, our findings are of potential interest, as they suggest that the clinical course of this phenotype might be influenced by endocrine factors.

Use of the TMA technique for biomarker studies in melanoma has several limitations, not least related to technical difficulties to obtain qualitative tissue cores from small lesions, thereby creating a selection bias towards larger tumours available for analysis. The majority of melanomas in our study were however thinner than 1 mm, also in the TMA cohort, and Ki67 remained an independent predictor of an impaired DFS and OS in men even after adjustment for tumour thickness, thus supporting its prognostic value also in thinner melanomas. Another potential limitation to the TMA technique is that heterogenously expressed markers might not be reliably determined. The only investigative biomarker in this study, Ki67, has been demonstrated to show variable expression in melanoma, depending on the growth phase [33]. We did however check for staining heterogeneity of Ki67 expression by comparing full-face sections and TMA cores from a subset of the tumours and found no obvious difference.

Another limitation to our study is that the comparatively low number of recurrences did not allow for a meaningful analysis of sex-related differences in the predilection of local, regional or distant recurrence. However, while the recurrence rate did not differ significantly between the sexes, the finding of a significantly prolonged survival in women compared to men after the first recurrence is consistent with previous studies [14].

Some of the findings in this study should be interpreted with caution, since they represent results from post hoc subgroup analyses of small numbers of patients. Therefore, these data need to be validated in larger patient cohorts. It should also be pointed out that since mean age at diagnosis was 65 years for women, interpretations relying on sex as evidence for influence of sex hormones must be made with caution until further knowledge has been gained. To this end, future studies are warranted to explore the effects of e.g. hormone replacement therapy on clinicopathological factors, molecular correlates and survival from melanoma.

## Conclusions

The prognostic value of tumour location, ulceration and Ki67 expression in melanoma seems to differ according to gender. These findings need to be validated in future studies, as they might help improve prognostication of patients with primary melanoma. Moreover, our data further support the value of sex-stratified analyses in biomarker studies.

## Abbreviations

DFS, Disease free survival; MSS, Melanoma specific survival; OS, Overall survival; HR, Hazard ratio; MDCS, Malmö Diet and Cancer Study; LMM, Lentigo maligna melanoma; SSM, Superficial spreading melanoma; NMM, Nodular malignant melanoma.

## Competing interests

The authors declare that no competing interests exist.

## Authors’ contributions

MF analysed the IHC staining, performed statistical analyses and drafted the manuscript, BN performed and analysed the IHC staining and helped draft the manuscript, LJ and JB collected clinical data, KJ conceived of the study, participated in its design and coordination and helped to draft the manuscript. All authors read and approved the final manuscript.
